# Using ex vivo culture to assess dynamic phenotype changes in human prostate macrophages following exposure to therapeutic drugs

**DOI:** 10.1038/s41598-021-98903-y

**Published:** 2021-09-29

**Authors:** Clovis Boibessot, France-Hélène Joncas, Aerin Park, Zohra Berrehail, Jean-François Pelletier, Typhaine Gris, Alain Bergeron, Paul Toren

**Affiliations:** 1 Laboratoire d’Uro-Oncologie Expérimentale, Centre de recherche du CHU de Québec-Université Laval, Axe Oncologie, 10 McMahon, rm 0877, Quebec, QC G1R 3S1 Canada; 2 Centre de recherche sur le Cancer de l’Université Laval, Quebec, Canada; 3 Département de chirurgie, Université Laval, Quebec, Canada

**Keywords:** Immunology, Innate immune cells, Tumour immunology, Cancer microenvironment, Cancer, Prostate cancer

## Abstract

Within the prostate tumor microenvironment (TME) there are complex multi-faceted and dynamic communication occurring between cancer cells and immune cells. Macrophages are key cells which infiltrate and surround tumor cells and are recognized to significantly contribute to tumor resistance and metastases. Our understanding of their function in the TME is commonly based on in vitro and in vivo models, with limited research to confirm these model observations in human prostates. Macrophage infiltration was evaluated within the TME of human prostates after 72 h culture of fresh biopsies samples in the presence of control or enzalutamide. In addition to immunohistochemistry, an optimized protocol for multi-parametric evaluation of cellular surface markers was developed using flow cytometry. Flow cytometry parameters were compared to clinicopathological features. Immunohistochemistry staining for 19 patients with paired samples suggested enzalutamide increased the expression of CD163 relative to CD68 staining. Techniques to validate these results using flow cytometry of dissociated biopsies after 72 h of culture are described. In a second cohort of patients with Gleason grade group ≥ 3 prostate cancer, global macrophage expression of CD163 was unchanged with enzalutamide treatment. However, exploratory analyses of our results using multi-parametric flow cytometry for multiple immunosuppressive macrophage markers suggest subgroup changes as well as novel associations between circulating biomarkers like the neutrophil to lymphocyte ratio (NLR) and immune cell phenotype composition in the prostate TME. Further, we observed an association between B7–H3 expressing tumor-associated macrophages and the presence of intraductal carcinoma. The use of flow cytometry to evaluate ex vivo cultured prostate biopsies fills an important gap in our ability to understand the immune cell composition of the prostate TME. Our results highlight novel associations for further investigation.

## Introduction

Prostate cancer (PCa) represents the most frequent non-cutaneous solid tumor and a leading cause of cancer death in men^1^. Androgen deprivation therapy (ADT) remains a mainstay of treatment, being used with radiation for higher risk localized disease as well as in all patients with recurrent or metastatic disease. Nonetheless, all men receiving ADT will eventually stop responding as the cancer develops to become castration resistant prostate cancer (CRPC), which is consistently lethal^2^.

Treatments for CRPC are expanding and it is increasingly important to understand patient and tumor biology to select biologically rationale and efficacious monotherapies and combination therapies^3^. In the last decade, several second line anti-androgen drugs have been discovered (e.g. abiraterone, enzalutamide, apalutamide and darolutamide), while chemotherapies such as docetaxel and cabazitaxel remain standard treatments. Immune checkpoint inhibitors and PARP inhibitors are under evaluation in combination clinical studies^4–6^. Moreover, these therapies are increasingly being advanced earlier in the course of treatment, including castration sensitive PCa^7^.

There is increasing evidence that immune cells within the tumor microenvironment (TME) contribute significantly to PCa progression. The prostate immune TME is preferentially enriched with myeloid cells compared to lymphocytes in both human and murine models^8–10^. Tumor-associated macrophages (TAMs), which can represent up to 30% of total immune infiltrating cells in prostate tumors, are characterized by immunosuppressive macrophage markers^11^ which can drive therapeutic resistance and promote tumor escape, metastasis, invasion, tissue remodeling and epithelial to mesenchymal transition^9,11–13^. In general, a higher density of macrophages in the tumor-containing prostate is associated with a poorer prognosis and worse overall survival^11,14^. Prior research also suggests that the proportion of CD206^+^ macrophages is higher in metastatic PCa compared to localized PCa^15^. Indeed, higher expression of M2 macrophage markers CD163 and CD206 in localized tumors is associated with an increased risk of metastasis^8,16,17^. Therefore, a better understanding of how TAMs respond to current therapies may lead to identification of prognostic and predictive biomarkers.

Current preclinical models are inadequate to reflect the importance of intra-tumor heterogeneity and the critical reciprocal interactions between the tumor cells and surrounding microenvironment, notably the interaction with the immune compartment. Since macrophages are a major immune component within the prostate TME, and since they have an important impact on PCa outcomes, we sought to evaluate how they are modulated by changes in the microenvironment using short-term ex vivo cultures of fresh prostate biopsies. In particular, following development of flow cytometry methods for individual phenotype analysis, we evaluated changes following treatment with enzalutamide.

## Results

### Ex vivo treatment of prostate biopsies with enzalutamide increases the CD163^+^ macrophage population

**Figure 1:**
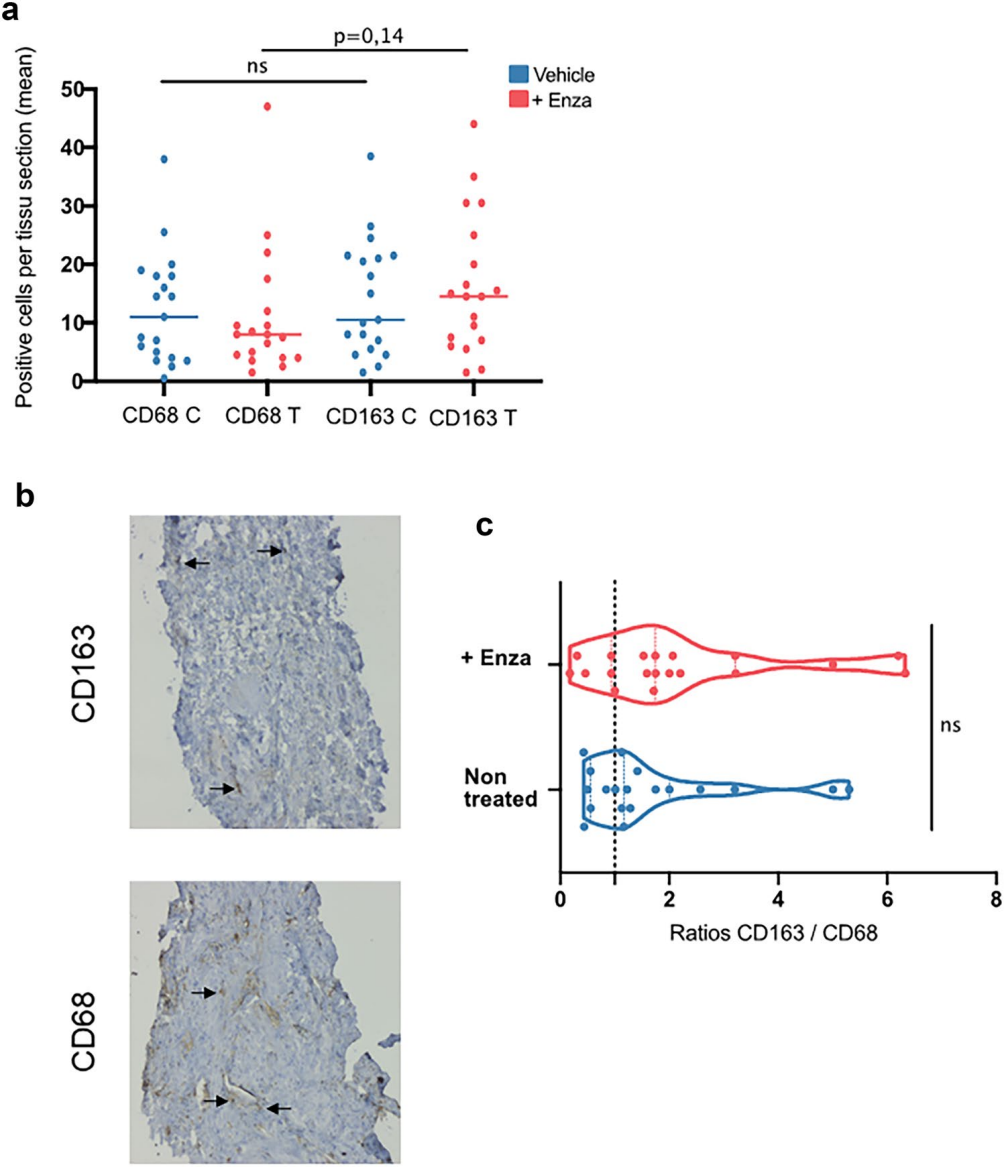
Effect of enzalutamide on the ratio of CD163^+^/CD68^+^ cells in ex vivo cultured prostate tumor specimens. Fresh prostate tumor needle biopsies were obtained cut in two halves and either treated with 10 µM enzalutamide (T) or vehicle as control (C) for 72 h. Biopsies were then washed and fixed in formalin and paraffin embedded. Tissue sections were prepared and tested with monoclonal antibodies (mAbs) against CD163 and CD68. Means of the number of CD163^+^ and CD68^+^ cells per biopsy were determined (**a**). Immunohistochemistry examples for both mAbs are shown (**b**), with the ratio of CD163^+^/CD68^+^ cells for each condition also shown (**c**), *n* = *19.*

We first assessed whether ex vivo treatment of PCa biopsies with enzalutamide could influence the phenotype of macrophages found in the prostate of men with PCa. Halves of five biopsies were cultured together for 72 h on surgifoam pads in standard medium culture in presence of 10 μM enzalutamide or vehicle control. At the end of the treatment, biopsies were fixed stained for the pan-macrophage marker CD68 or CD163, a marker of M2-polarized macrophages. The mean of CD68^+^ and CD163^+^ positive cells was determined for the two conditions and the ratio of CD163^+^/CD68^+^ cells was calculated for each patient series of 5 biopsies. Staining results for each marker and condition are shown in Fig. 1a. Normalizing CD163 expression by CD68 expression for each treatment to control pair demonstrated a slight increase of CD163^+^/CD68^+^ cells with enzalutamide versus control (Fig. 1b,c).

### Ex vivo treatment of PCa biopsies with enzalutamide induces no changes in macrophage markers in whole immune cell or macrophage populations

**Figure 2:**
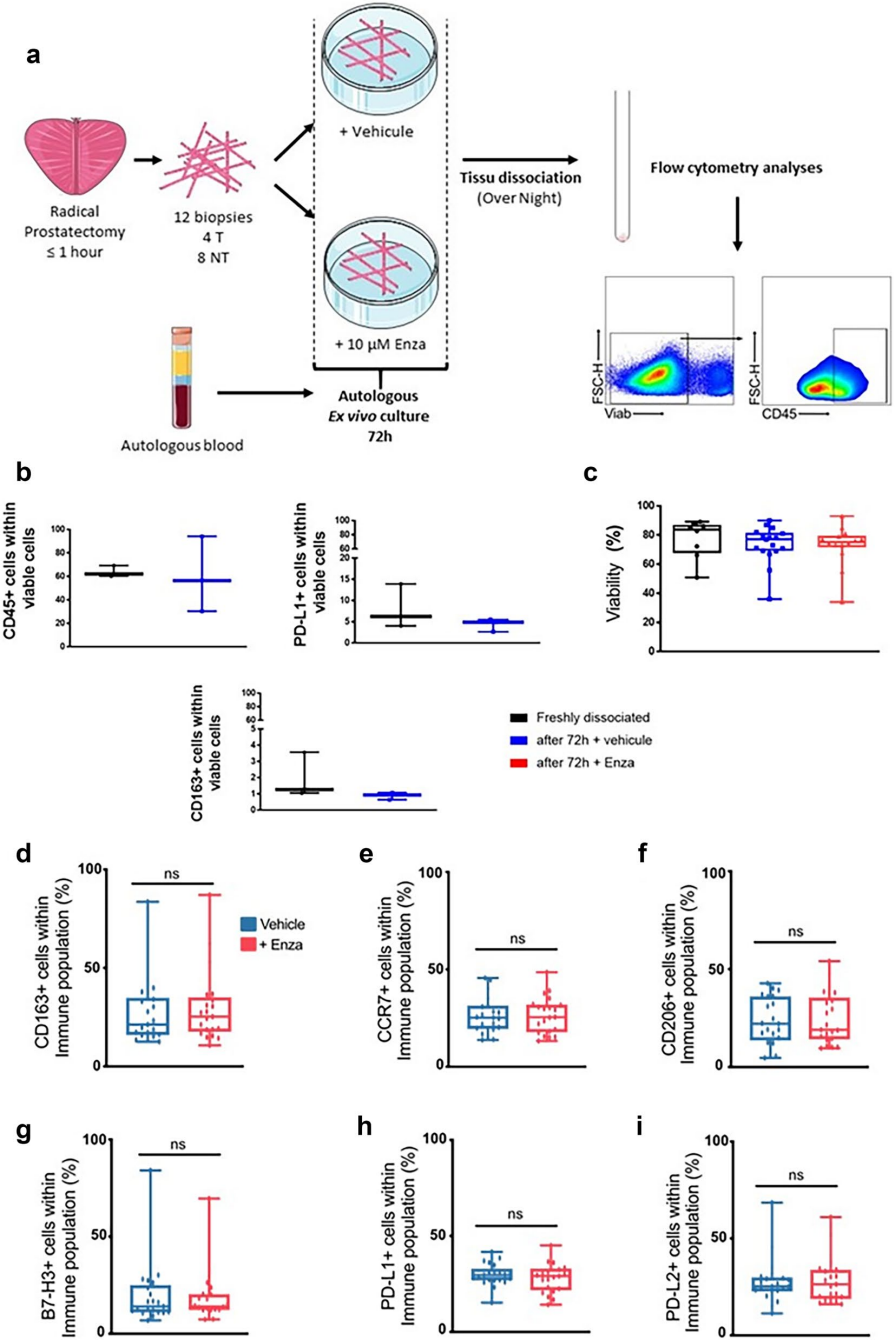
Ex vivo short-term culture and treatment of prostate cancer biopsies with enzalutamide A schematic of biopsy processing and gating strategy for CD45 immune cells is shown (**a**). Comparison of CD45, PD-L1 and CD163 expression of paired biopsies freshly dissociated and after 3 days of culture (n = 3) (**b**). Comparison of cell viability in freshly dissociated biopsies (n = 8) and treated or control biopsies cultured for 72 h (n = 14) (**c**). Evaluation of M1-associated marker (CCR7), M2-associated marker (CD163, CD206) and immune checkpoints (B7-H3, PD-L1, and PD-L2) within total immune cell population between treated and control-treated paired-samples (**d**–**i**). n = 21, paired and non-paired Student’s T-test, ns = statistically not significant.

To circumvent the limitations of immunohistochemistry on ex vivo cultured biopsies, we next sought to develop methods using multicolor flow cytometry. Moreover, to avoid the possibility of complement activation altering immune cell phenotype, we used autologous serum instead of fetal bovine serum (FBS) (Fig. 2a). As previously observed for immunohistochemistry^18^, a comparison of immune cell marker expression by flow cytometry between paired immediately processed biopsies and those cultured for three days did not show significant differences (Fig. 2b). Similarly, no significant decrease in viability was observed after three days of culture ex vivo with control or enzalutamide treatment (Fig. 2c). We next optimized parameters for a panel of 10 monoclonal antibodies (mAbs) to evaluate phenotypic markers on macrophages (Supplementary Table S1). This panel was used for the final 21 patients in the second cohort (Supplementary Table S2).

In concurrent research on the prognostic role of macrophages in PCa, we obtained evidence that CD163^+^ cells localized in greater numbers in peri-tumoral areas have a stronger prognostic value for long-term clinical outcomes and thus might have an important biological role. Therefore, for these final 21 patients we targeted four biopsies in the tumor area and eight biopsies in zones with negative systematic biopsies. We observed no significant difference in the proportion of cells expressing the M1-associated marker CCR7, the M2-associated markers CD163 and CD206 or either the immune checkpoints PD-L1, PD-L2 and B7-H3 between the enzalutamide-treated and non-treated biopsies (Fig. 2d–i).

**Figure 3:**
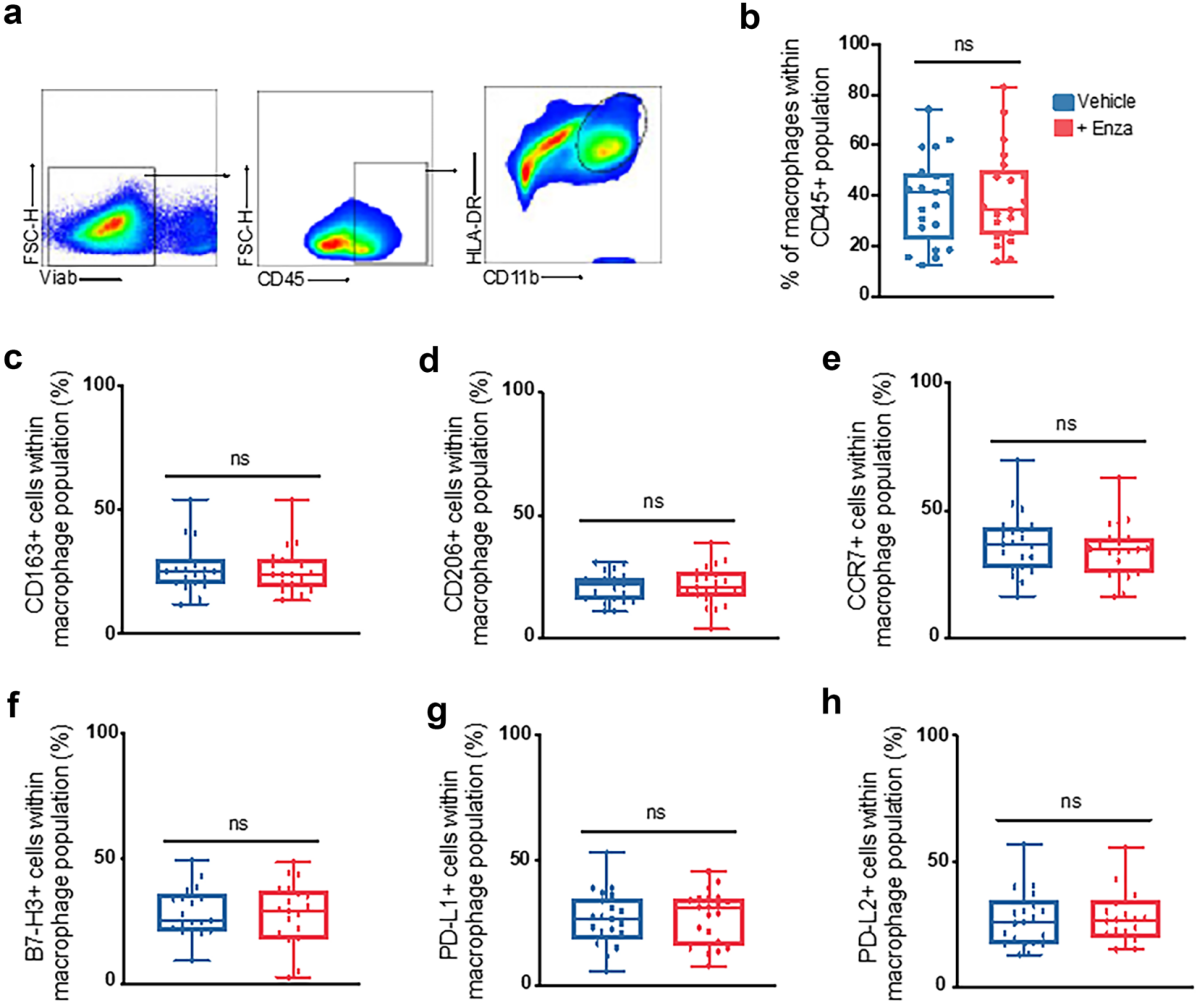
Enzalutamide treatment of ex vivo cultured prostate cancer biopsies induces no change in global macrophage phenotype. Gating strategy to evaluate macrophage population within CD45^+^ immune population (**a**). Evaluation of macrophage proportion between treated and control-treated paired samples (**b**). M1-associated marker (CCR7), M2-associated marker (CD163, CD206) and immune checkpoints (B7-H3, PD-L1, and PD-L2) between treated and control-treated paired-samples (**c**–**h**). *n* = 21, paired and non-paired Student’s T-test, ns = statistically not significant.

We next analyzed the expression of these markers within the macrophage population defined as CD45^+^/CD11b^+^/HLA-DR^+^ cells (Fig. 3a). The macrophage population represented about 40% of the CD45^+^ immune cells found in the biopsies (Fig. 3b). Again, when comparing between enzalutamide-treated and control biopsies, we found no significant difference in the proportion of cells expressing the 6 markers in the whole CD45^+^/CD11b^+^/HLA-DR^+^ macrophage population (Fig. 3c–h).

### Ex vivo treatment of PCa biopsies with enzalutamide induces changes in specific macrophage populations

**Figure 4:**
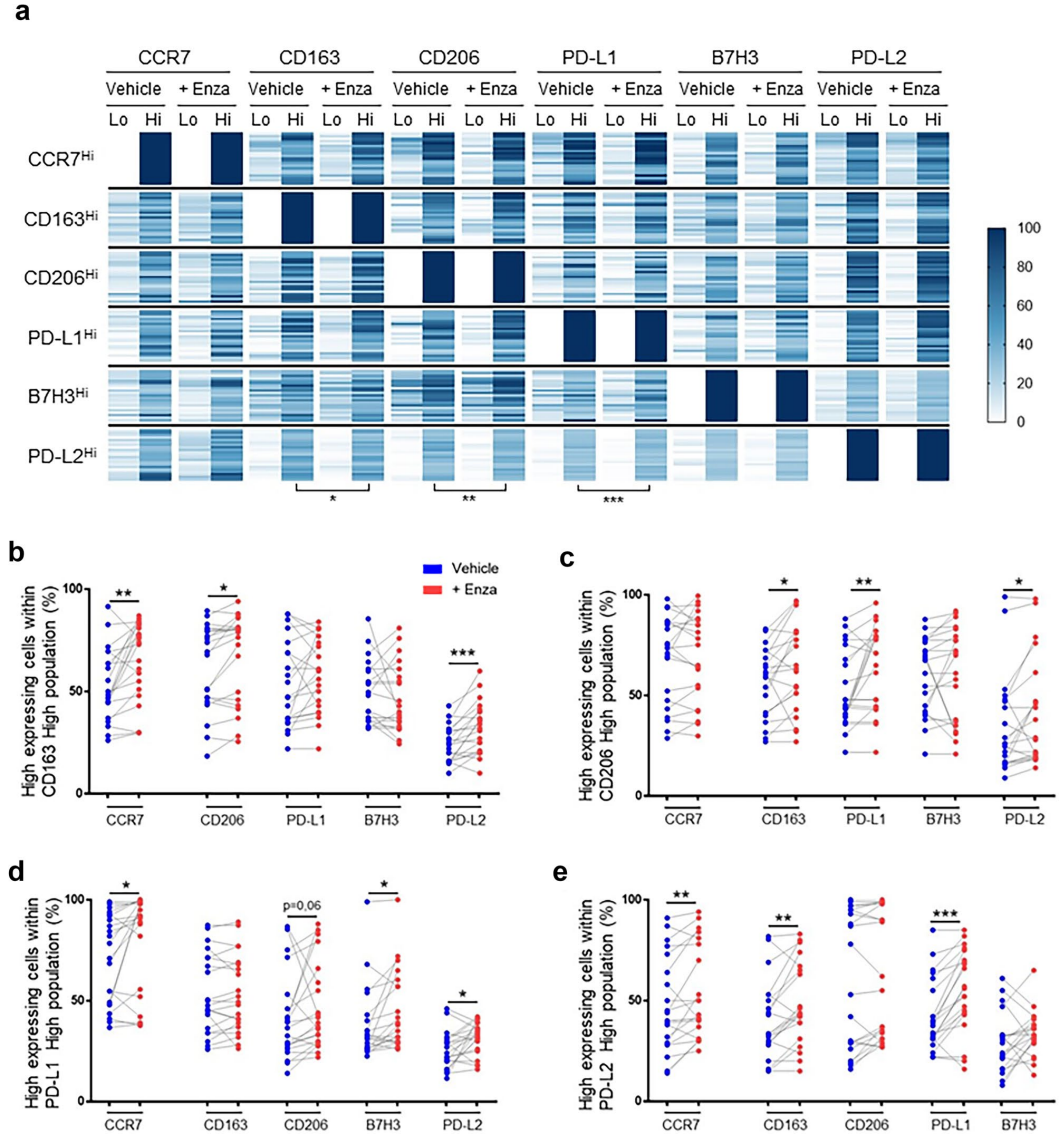
Enzalutamide treatment of prostate cancer biopsies induces changes in detailed macrophage landscape. For each patient, the expression of M1(CCR7) and M2 (CD163, CDD206, PD-L1, PD-L2) markers was quantified within macrophage (CD45^+^/CD11b^+^/HLA-DR^+^) clusters defined by manually gated high and low expression of indicated marker. Scale bar indicates frequency of high expression from 0 to 100% of the indicated markers. Overall, there were significant increases in CD163, CD206 and PD-L1 expression across all high-expressing marker macrophage clusters as summarized in a heatmap. (**a**). Details of changes in high-expressing CD163-, CD206-, PD-L1- and PD-L2- macrophages clusters for paired enzalutamide-treated and control samples (**b**–**e**). n = 21, paired and non-paired Student’s T-test, *p < 0.05, **p < 0.005, ***p < 0.0005.

To further analyze the effect of enzalutamide on the macrophages and to account to individual variability, we manually divided the global CD45^+^/CD11b^+^/HLA-DR^+^ macrophage population according to high and low expression of the markers for each patient, with a heatmap demonstrating the expression of concomitant markers within each high/low marker macrophage population according to patient biopsy control/treatment pairs. (Fig. 4a). We observed that in the CD163^hi^ macrophage population enzalutamide treatment induced an increase in the proportion of cells concomitantly expressing high levels of the CD206 M2-associated marker but also the CCR7 M1-associated marker as well as the immune checkpoint PD-L2 (Fig. 4b). In the CD206^hi^ macrophage population, we also observed an increase in the percentage of cells concomitantly expressing high levels of CD163, PD-L1 or PD-L2 after treatment with enzalutamide (Fig. 4c). When focusing on the PD-L1^hi^ macrophage population, treatment with enzalutamide induced a significant increase in the proportion of CCR7^hi^, B7-H3^hi^ and PD-L2^hi^, with a similar non-significant increase in CD206^hi^ co-expressing cells (Fig. 4d). In PD-L2^hi^ macrophages, enzalutamide induced an increase in the proportion of cells co-expressing CCR7, CD163 and PD-L1 at high levels (Fig. 4e).

### Association between the macrophage-associated markers and clinical parameters

To better assess the impact of changes observed within the macrophage populations, we next explored associations between the proportion of macrophages expressing CD163, PD-L1, PD-L2, B7-H3, CD206 and CCR7 within the different immune cell populations for each patient and available clinicopathological data. These analyses showed several associations with clinical variables. Firstly, there was an association between the presence of inflammatory CCR7 expression and favourable tumor characteristics. Within the global CD45^+^ immune cell population, CCR7^+^ population correlated with smaller prostate tumor volume (PTV) (r = − 0.507, p = 0.026) and less extra-prostatic extension (EPE) (r = − 0.517, p = 0.023) (Supplementary Table S3). In terms of mean fluorescence intensity (MFI), higher CCR7^+^ macrophage (CD45^+^/CD11b^+^/HLA-DR^+^) expression correlated with lower frequency of positive surgical margins (r = − 0.467, p = 0.043) (Supplementary Table S4).

Our second observation was that anti-inflammatory markers often correlated with inflammatory markers. Indeed, the strongest correlation observed was the proportion of PD-L1^+^ macrophages with the neutrophil to lymphocyte ratio (NLR) (a systemic inflammatory marker) (r = 0.748, p = 0.001) (Table 1). Similarly, both the MFI of PD-L1^+^ (r = 0.594, p = 0.007) and CCR7^+^ (r = 0.484, p = 0.035) macrophages positively correlated with NLR (Supplementary Table S4). Among macrophages, the CCR7 MFI was positively correlated with PD-L1 intensity (r = 0.675, p = 0.002) and CD163 (r = 0.496, p = 0.031). Thus, while not inflammatory, the proportion of PD-L1^+^ or CD206^+^ macrophages also correlated with smaller PTV (r = − 0.503, p = 0.027; r = − 0.513, p = 0.024). Further, the proportion of HLA-DR^+^ or CD163^+^ cells both correlated with less EPE (r = − 0.528, p = 0.02, r = − 0.517, p = 0.023) (Supplementary Table S3).

**Table 1:** Correlation between clinico-pathological characteristics and macrophage-associated markers within the macrophage population.

	CD45/CD11b/HLA-DR^+^	CD45/CD11b/HLA-DR/CD163^+^	CD45/CD11b/HLA-DR/B7-H3^+^	CD45/CD11b/HLA-DR/CD206^+^	CD45/CD11b/HLA-DR/PD-L1^+^	CD45/CD11b/HLA-DR/PD-L2^+^	CD45/CD11b/HLA-DR/CCR7^+^
NLR	0.312	**0**.405*	0.147	0.277	**0**.748***	0.002	0.103
PSA	− 0.194	− 0.211	− 0.135	− 0.294	− 0.436	0.081	0.129
EPE	− **0**.597**	− 0.298	0.179	− 0.358	− 0.298	0.308	− 0.338
SVI	− 0.421	0.039	0.158	− 0.131	0	0.395	0.079
IDC	− 0.154	− 0.279	**0**.481*	− 0.230	− 0.105	− 0.086	− 0.154
PNI	− 0.425	− 0.278	0.294	− 0.229	− 0.360	0.425	0.032
LVI	− 0.387	− 0.086	0.215	0.258	0	0.172	0.215
PTV	− 0.372	− 0.292	0.229	− **0**.513*	− **0**.503*	− 0.019	− 0.391
PM	− 0.214	− 0.282	− 0.038	− 0.272	− 0.214	− 0.116	− 0.428

Spearman's rank correlation table showing the correlation between clinico-pathological characteristics and the proportion of cells expressing high levels of M1-associated marker (CCR7), M2-associated markers (CD163, CD206) or immune checkpoints (B7-H3, PD-L1 or PD-L2) within the macrophage (CD11b^+^ HLA-DR^+^ CD45^+^) population. Spearman’s rank correlation coefficients are presented below. Correlation coefficients (r_s_) > 0.3 or < − 0.3 with significant p-values are highlighted in bold.

*NLR* Neutrophil-to-lymphocyte ratio, *PSA* Prostate specific antigen, *EPE* Extraprostatic extension, *SVI* Seminal vesicle invasion, *IDC* Intraductal carcinoma of the prostate, *PNI* Perineural invasion, *LVI* Lymphovascular invasion, *PTV* Prostate tumor volume, *PM* Positive margin.

*p ≤ 0.05, **p ≤ 0.01, ***p ≤ 0.001.

Thirdly, we observed a correlation between some immunosuppressive markers. Among macrophages, PD-L1 MFI was associated with higher levels of B7–H3 (r = 0.461, p = 0.047) and CD206 (r = 0.707, p = 0.001) (Supplementary Table S3). Similarly, CD206 MFI was associated with higher PD-L2 MFI (r = 0.768, p < 0.001) (Supplementary Table S3).

In contrast to correlations among the macrophage populations, the overall proportion of CD11b^+^ cells correlated with higher PTV (r = 0.479, p = 0.037) (Supplementary Table S3). We also observed a positive correlation between B7-H3 expression and intraductal carcinoma of the prostate (r = 0.481, p = 0.037). No correlation of any immune cell parameters with pre-operative PSA values was observed.

## Discussion

An understanding of the biology of individual tumors is central to personalizing treatment. Our study presents important data on individual immunologically analyses of fresh patient prostate tumors. We highlight the feasibility to evaluate dynamic changes in phenotypic markers of macrophages from human surgical specimens. Further, we identify novel associations with clinicopathologic study for further investigation.

Using short-term ex vivo culture of tumor biopsies as a novel method to assess the effect of exposure of therapeutic drugs on the immune TME, we did not observe that enzalutamide impacted the global immune cell phenotype but did effect relative changes on macrophage polarization. While our preliminary observations using IHC of macrophage infiltrates suggested enzalutamide may exert changes in CD68^+^ and CD163^+^ cell populations, this approach proved both laborious and to have a limited sensitivity^19^. Nonetheless, immunohistochemistry is one of the most-reported techniques for protein phenotyping of ex vivo cultured tumor samples^20,21^. Given the superior multiparametric evaluation possible with flow cytometry, we sought to use this more quantitative approach for similar measurements of dynamic changes induced by treatment of patient biopsy specimens. With CD163 expression associated with poor prognosis in other PCa studies^16^, we were interested to further evaluate changes induced by enzalutamide in TAMs.

The direct experimental evaluation of specific cells from within tumors represents a major strength of using ex vivo studies of patient samples, but are the results obtained representative of in vivo biology? Prior work supports that ex vivo cultured prostate tumors retain tumoral characteristics^18,20^. Tissue dissociation techniques for flow cytometry permit accurate and detailed measurements of individual cells, though tissue heterogeneity must always be considered. The gold standard for treatment-induced changes observed in ex vivo studies remains uncertain but is perhaps information obtained from serial biopsies from the same patient tumor following treatment (evidently difficult to obtain). In our study, the correlations between specific tumor immune cell counts from concurrently obtained complete blood counts provide indirect support that the results are representative of human tumor biology. That all changes in macrophage subpopulations we observed were only increases supports that these were true biologic changes. However, no direct evaluation of tumor cells was performed, such as assessment for downregulation of the AR-axis expected to occur following enzalutamide. Further research evaluating different cell populations as well as different treatments using prostate and other cancer samples is important to further establish the validity of evaluating dynamic changes induced by treatment with ex vivo cultures.

Defining macrophage phenotypes is complex, with a M1 and M2 dichotomy commonly used as a simplification for reference to an inflammatory and anti-inflammatory end of a phenotype spectrum. Following prior experiments and the literature, we focused on CCR7 as an inflammatory, M1 marker. Confirming an inflammatory, anti-tumoral association, the proportion of M1-associated CCR7^+^ cells in the whole CD45^+^ immune cell population was shown to be correlated with low tumor volume and absence of extraprostatic extension. In addition, when analyzed within the macrophage populations, CCR7 intensity was associated with higher PD-L1 and CD163 intensity. Cell surface expression of CCR7, receptor for CCL19 and CCL21, was shown to be associated with CCL19/CCL21-mediated in M1 macrophage chemotaxis^23^. In our study, we noticed that both immune checkpoints and M2-associated markers and CCR7 were associated within the macrophage population. This could be related to the increase of macrophage recruiting within the prostate and their conversion into TAMs. Several studies have reported potential targeting strategies against TAMs^22,24,25^. Our results suggest that the CCL19/CCL21-CCR7 axis might be a potential therapeutic target to decrease macrophage recruitment within the prostate TME, as proposed in other cancer sites^26^.

Our correlation analyses between flow cytometry and clinical parameters (Table 1) had several interesting findings. We found that both a higher proportion of PD-L1^+^ in macrophages as well as a higher PD-L1 intensity significantly associated with a higher neutrophil:lymphocyte ratio (NLR). In PCa patients a lower NLR was previously associated with better outcomes in patients with localized or metastatic disease^27–29^. Our results also provide the first evidence that NLR may be a proxy for the immune infiltrate present within prostate tumors. This raises questions whether the increased NLR observed during treatment^27^ is also a proxy for increased concomitant immunosuppression within the PCa TME. This possibility is supported by our observation that PD-L1 expression was higher in macrophages expressing high levels of CD206, CCR7 or PD-L2. Another interesting finding was that a higher proportion of B7-H3^+^ macrophages was associated with the presence of aggressive intraductal carcinoma (IDC). Higher levels of B7-H3 were also correlated with higher levels of PD-L1 in macrophages which themselves correlate with elevated NLR. Together, these observations suggest the utility of multiparametric immunophenotyping of PCa macrophages compared to single-marker evaluation studies.

While globally no significant changes in macrophage markers was observed after 3 days of enzalutamide treatment, we did observe certain trends in macrophage sub-populations. While exploratory in nature, these analyses suggest that enzalutamide may alter the phenotype of certain macrophages over time. Notably, the changes we observed all occur among macrophages highly expressing immunosuppressive markers (i.e. CD163, CD206, PD-L1 and PD-L2). While not all changes were statistically significant, the overall tendency was for increases in both immunosuppressive and inflammatory (i.e. CCR7) markers following enzalutamide treatment. Interestingly, this dual increase in both M1 and M2 markers is consistent with our above observations regarding correlations with NLR, with an elevated NLR following enzalutamide treatment associated with poorer response to enzalutamide^30^. Together, this suggests the possibility that changes in macrophage subpopulations following enzalutamide may contribute to treatment resistance.

The number of patients in our study is a limitation, but even so we were able to associate some clinical outcomes with specific macrophage phenotypes. As an exploratory study, adjustment for multiple testing was not performed. Further, longer-term clinical outcomes with a larger patient cohort will help to validate the observed clinical associations with different immunophenotypes. Although other groups have published protocols for assessing tumor response to therapeutics ex vivo, these generally involve tumor manipulation (e.g. by sectioning) prior to ex vivo culture^20,21^. The limitation of this technique lies in the partial knowledge of the tumor area location in the biopsy. Compared to divided tissue sections, the use of multiple biopsies pooled together in our protocol decreases manipulations and simplifies rapid culture of fresh tissue while providing broader sampling, though the histology is not observable^31^. Finally, while prior studies^18^ do not suggest significant marker changes occur during 72 h of ex vivo culture, the intra-patient comparisons in our study were limited and the possibility that marker changes occur due to ex vivo culture or dissociation techniques cannot be excluded.

In summary, we show that ex vivo treatment of prostate biopsies combined with flow cytometry analysis has considerable potential to reveal macrophage phenotypes associated with clinical outcomes. This technique allows a better and more complete analysis of the immune cell infiltrates, including dynamic changes induced by treatments. Thus, this protocol could be adapted for testing various therapies to better understand treatment-induced changes in the infiltrating immune cells, potentially revealing clinically useful biomarkers or mechanisms of resistance.

## Materials and methods

### Prostate biopsy procurement

Fresh prostate biopsies were obtained from men undergoing radical prostatectomy at Centre Hospitalier Universitaire de Québec, L’Hôtel-Dieu de Québec Hospital (QC, Canada) between 2017 and 2020. The first cohort included 19 men with Gleason Grade Group (GGG) ≥ 2 PCa on pre-operative biopsy, while the second cohort included 29 men with GGG ≥ 3 on pre-operative biopsy. Informed consent was obtained to participate to the institutional uro-oncology biobank (URO-1). The study was approved by the research ethics committee of the Centre Hospitalier Universitaire de Québec-Université Laval (#2012-1002, #2019-4181), in accordance with the Declaration of Helsinki. Within 1 h of surgical removal, the prostate was brought to the pathology department where typically twelve 18-gauge needle biopsies were taken under supervision of a pathologist.

### Short-term culture of ex vivo cultured prostate biopsies

For the first cohort, biopsies were cultured according to a previously described protocol^18^. Briefly, five fresh biopsies were washed in HBSS, then divided in half and distributed between two petri dishes where they were placed on Surgifoam (Johnson & Johnson, New Brunswick, NJ, USA) soaking in EMEM media (Wisent Bioproducts, St-Bruno, QC, Canada) supplemented with Gibco antibiotic–antimycotic (Thermo Fisher Scientific, Ottawa, ON, Canada) and 10% (FBS) (Wisent Bioproducts). Culture was performed at 37 °C with 5% CO_2_. Paired halves were cultured for 72 h in media added with 10 μM enzalutamide or vehicle (dimethyl sulfoxide, Fischer Chemical), as control. After treatments, biopsies were fixed in formalin and embedded in paraffin.

For the second cohort, a new protocol using autologous serum instead of FBS was developed to better reproduce in situ conditions. Sextant zones involved with tumor on pre-prostatectomy biopsy results were used to identify tumor location, with magnetic resonance imaging (when available) and palpation of the surgical specimen used for confirmation. For each fresh prostate, biopsies were washed in HBSS without Ca^2+^ Mg^2+^, distributed evenly in two petri dishes and then cultured for 72 h in freshly prepared complete autologous media. Complete medium consists of Advanced DMEM-F12 media supplemented with 100 mg/L of antimicrobial agent Primocin (InvivoGen, San Diego, CA, USA), 10 mL/L of Glutamax (ThermoFisher scientific, #35050061), 2978 mg/L of HEPES (Sigma-Aldrich), with 10% autologous serum. One half of biopsy specimens were cultured in presence of 10 μM enzalutamide and the other half with vehicle control. At the end of the incubation period, the biopsies from each condition were pooled and dissociated for cell analysis in multicolor flow cytometry.

### Immunohistochemistry

Formalin-fixed paraffin-embedded prostate tumor biopsies were cut into 5 μm thick sections and dried overnight at 37 °C. Sections were deparaffinized, and heat induced antigen retrieval was performed for 20 min at 92 °C using the PT Link, Pre-Treatment Module for Tissue Specimens (Dako, Burlington, ON, Canada) with either citrate buffer pH 6.1 for CD68 (Dako, Code K8005: EnVision™ FLEX, Low pH) or Tris/EDTA, pH 9 for CD163 (Dako, Code K8004: EnVision™ FLEX, High pH). Endogenous peroxidase activity was blocked by incubation in 3% peroxide solution for 10 min. Staining was performed using the IDetect Super Stain System (HRP) (ID labs, London, ON, Canada) as follow. First slides were incubated for 10 min at room temperature with Super block solution to block nonspecific background staining. Then, incubation with primary antibody was carried out for 1 h at room temperature with a monoclonal antibody (mAb) against CD163 (clone 2G12, dilution 1:2000, Abcam, Toronto, ON) or against CD68 (clone KP1, dilution 1:400, Abcam). After washes, slides were incubated for 10 min with biotinylated polyvalent secondary antibody and 10 min with biotin-HRP. DAB staining was achieved by a 5 min incubation. Finally, slides were rinsed, counterstained with hematoxylin, dehydrated and mounted with coverslip using MM 24 low viscosity mounting medium (Leica Microsystems, Durham, USA). The number of CD68^+^ and CD163^+^ cells in each of the biopsy halves were counted by 2 independent readers. Biopsies with no CD68^+^ or CD163^+^ cells were excluded. To compare results between biopsies, the ratio of CD163^+^/CD68^+^ cells was used. Paired biopsy halves (individually divided between control vs treatment) allowed comparison of treatment effect between patients.

### Tissue dissociation

Biopsies were washed two times with HBSS with Ca^2+^ Mg^2+^, then incubated overnight at 37 °C with 5% CO_2_ in complete medium without autologous serum. Media was supplemented with Type II collagenase (Thermo scientific, #17101015, final concentration of 300 U/mL) and 2 U/mL of DNase (Sigma #10104159001). The day after, dissociated biopsies were washed with HBSS with Ca^2+^ Mg^2+^ then incubated with 1 mL of Accutase (Corning, #25-058-ci) per 6 biopsies for 20 min at 37 °C. Dissociated cells were then collected, washed, stained and filtered for multicolor flow cytometry analyses.

### Cell staining and flow cytometry analyses

Multiparameter flow cytometry analyses were performed on fresh samples. Compensation controls were done using compensation beads (BD CompBeads, BD Biosciences, #552843) and fluorescence minus one (FMO) control strategy on fresh samples to identify gating boundaries. Cells were incubated with Seroblock (BIO-RAD, #BUF070B) for 5 min, and then with a cocktail of mAbs against CD11b, HLA-DR, CCR7, CD163, CD206, PD-L1 and B7-H3 (see Supplementary Table S1). Stained cells were acquired using a BD LSRFortessa cytometer (BD Biosciences, Immunocytometry Systems, San Jose, CA, USA) and data collection was obtained using BD FACS Diva software (BD Biosciences). Data analysis was performed using FlowJo software (v10.5.2, Flowjo, LLC). Cell viability was analyzed using the BD Horizon fixable viability stain (FVS-780, BD Biosciences) or FVS-450 (BD Biosciences) and doublets were excluded based on forward scatter-A against forward scatter-H.

### Statistical analyses

Specimen biopsy normality results were evaluated by both D’Agostino & Pearson normality test and Shapiro–Wilk normality test using GraphPad Prism 8.0 (Graphpad Software, San Diego, CA, USA). For nonparametric distribution, unpaired Mann–Whitney test or paired Wilcoxon matched pairs signed rank test were used. For parametric distributions, paired and unpaired Student’s T-test was used. Correlation between parameters was assessed using Spearman correlation coefficients.

## Supplementary Information

Supplementary Information.
