# Supplementary material for: Using ex vivo culture to assess dynamic phenotype changes in human prostate macrophages following exposure to therapeutic drugs
Source: Sci Rep. 2021 Sep 29;11:19299. doi: 10.1038/s41598-021-98903-y (PMC8481239; doi:10.1038/s41598-021-98903-y)
Supplement: Supplementary file 1 — Supplementary Information. [file 41598_2021_98903_MOESM1_ESM.pdf]

Supplemental Results

Using ex vivo culture to assess dynamic phenotype changes in human prostate macrophages following exposure to therapeutic drugs

Clovis Boibessot[1,2], France-Hélène Joncas[1,2], Aerin Park[1,2], Zohra Berrehail[1,2], Jean-François Pelletier[1,2], Typhaine Gris[1,2], Alain Bergeron[1,2,3] and Paul Toren[1,2,3].

[1]Laboratoire d'Uro-Oncologie Expérimentale, Centre de recherche du CHU de Québec-Université Laval, Axe Oncologie, Québec; [2]Centre de recherche sur le cancer de l'Université Laval, Québec; [3] Département de chirurgie, Université Laval.

Contents:

Supplemental Table 1. Antibodies for multicolor flow cytometry analyses.

Supplemental Table 2: Baseline characteristics of patients with fresh prostate biopsy samples evaluated for macrophages markers by flow cytometry following 72h of ex vivo culture

Supplemental Table 3: Correlation between clinico-pathological characteristics and macrophage-associated markers within the total immune cell population.

Supplemental Table 4: Correlation between clinico-pathological characteristics and mean fluorescence intensity of macrophage-associated markers within the macrophage population.

Supplemental Table 1. Antibodies for multicolor flow cytometry analyses.

Reactivity	Abs	Fluorochrome	Clone	Isotype	#Product	Provider
**Human**	CD11b	APC/Cy7	ICRF44	Mouse IgG1, κ	301342	Biolegend
**Human**	HLA-DR	PerCP-Cy 5.5	G46-6	Mouse IgG2a, κ	552764	BDBiosciences
**Human**	CD197	PE	3D12	Mouse IgG2a, κ	552176	BDBiosciences
**Human**	CD163	AF647	GHI/61	Mouse IgG1, κ	333620	Biolegend
**Human**	CD206	BV605	19,2	Mouse IgG1, κ	740417	BDBiosciences
**Human**	B7-H3	PE/Cy-7	MIH42	Mouse IgG1, κ	351007	Biolegend
**Human**	PD-L1	BB515	MIH1	Mouse IgG1, κ	564554	BDBiosciences
**Human**	PD-L2	BV421	MIH18	Mouse IgG1, κ	563842	BDBiosciences
**Human**	PD-1	PE-CF594	EH12.1	Mouse IgG1, κ	565024	BDBiosciences
**Human**	CD45	V500	515	Mouse IgG1, κ		
**Mammalian**	FVS-780	APC-Cy7			565388	BDBiosciences

Supplemental Table 2: Baseline characteristics of patients with fresh prostate biopsy samples evaluated for macrophages markers by flow cytometry following 72h of ex vivo culture. A total of 28 patients were evaluated in this manner, the latter 21 patients (final panel) were evaluated by the same panel of immunosuppressive macrophage markers (Suppl. Table 1).

	**Final panel (n=21)**	**All patients (n = 28)**
**Age (year)**	65,6 ± 5,1	65,2 ± 5,2
**Surgical Pathology Gleason Group Grade**		
2	6	8
3	11	15
4/5	4	6
**T Stage**		
pT2	8	11
pT3a	10	11
pT3b/pT4	3	4
**N Stage**		
Negative	14	18
Positive	7	8
**Margin**		
Negative	11	14
Postive	10	12
**Mean Prostate Tumor Volume (min – max)**	22,9 (1,5-60)	23,5 (1,5-60)
**Extraprostatic extension (EPE)**	13	15
**Intraductal carcinoma (IDC)**	10	11

Supplemental Table 3: Correlation between clinico-pathological characteristics and macrophage-associated markers within the total immune cell population.

Spearman's rank correlation table showing the correlation between clinico-pathological characteristics and the proportion of cells expressing high levels of the global macrophage-associated markers (CD11b, HLA-DR), M1-associated marker (CCR7), M2-associated markers (CD163, CD206) or immune checkpoints (B7-H3, PD-L1 or PD-L2) within the $CD45^+$ cell population. Spearman’s rank correlation coefficients are presented below. Negative correlations are presented in pale to dark green squares while positive correlations are presented from yellow to dark red squares. *Correlation coefficients (r_s) >0.3 or <-0,3 with significant p-values are highlighted in bold. * =p≤0,05, ** =p≤0,01, *** =p≤0,001. NLR=Neutrophil-to-lymphocyte ratio, PSA=Prostate specific antigen, EPE= Extraprostatic extension, SVI=Seminal vesicle invasion, IDC=Intraductal carcinoma of the prostate, PNI=Perineural invasion, LVI= Lymphovascular invasion, PTV= Prostate tumor volume, PM= Positive margin.*

	CD45+	CD45+/ HLA-DR+	CD45+/ CD11b+	CD45+/ CCR7+	CD45+/ CD163+	CD45+/ B7-H3	CD45+/ CD206	CD45+/ PD-L2	CD45+/ PD-L1
NLR	0.058	**0.688*** **	-0.147	0.061	0.145	0.364	0.324	0.135	-0.144
PSA	0.190	-0.38	**0.466***	-0.129	-0.043	-0.364	-0.436	0.243	0.211
EPE	-0.090	**-0.528***	0.278	**-0.517***	**-0.517***	-0.219	-0.338	-0.019	-0.01
SVI	**0.382***	-0.237	0.105	-0.289	-0.105	-0.079	-0.237	0.263	-0.052
IDC	-0.169	-0.163	0.134	0.019	-0.404	0.15	-0.288	-0.077	-0.333
PNI	0.264	-0.458	**0.523***	-0.261	-0.032	-0.098	-0.425	0.360	0.213
LVI	0.096	0.150	0.1721	-0.258	-0.387	0.172	0	0.043	-0.172
PTV	-0.104	-0.359	**0.479***	**-0.507***	-0.328	0.0097	-0.365	0.024	-0.088
PM	-0.206	-0.253	-0.214	-0.214	-0.408	-0.097	0.058	-0.311	-0.146

Supplemental Table 4: Correlation between clinico-pathological characteristics and mean fluorescence intensity of macrophage-associated markers within the macrophage population.

Spearman's rank correlation table showing the correlation between clinico-pathological characteristics and the mean fluorescence intensity (MFI)) of M1-associated marker (CCR7), M2-associated markers (CD163, CD206) or immune checkpoints (B7-H3, PD-L1 or PD-L2) in the macrophage ($CD11b^+$ $HLA\text{-}DR^+$ $CD45^+$) population. Spearman's rank correlation coefficients are presented below. Negative correlations are presented in pale to dark green squares while positive correlations are presented from yellow to dark red squares. *Correlation coefficients (r_s) >0.3 or <-0,3 with significant p-values are highlighted in bold. *=p≤0,05, **=p≤0,01, ***=p≤0,001. NLR=Neutrophil-to-lymphocyte ratio, PSA=Prostate specific antigen, EPE= Extraprostatic extension, SVI=Seminal vesicle invasion, IDC=Intraductal carcinoma of the prostate, PNI=Perineural invasion, LVI= Lymphovascular invasion, PTV= Prostate tumor volume, PM= Positive margin.*

	MFI (CD163)	MFI (PD-L1)	MFI (PD-L2)	MFI (CCR7)	MFI (B7-H3)	MFI (CD206)
NLR	-0,035	**0,594****	-0,191	**0,484***	0,417	0,098
PSA	0,042	-0,014	0,261	-0,226	-0,273	0,224
EPE	-0,398	-0,069	0,239	-0,338	0,298	0,259
SVI	0,052	0,316	0,289	0,237	0,342	0,421
IDC	-0,327	-0,057	-0,057	-0,173	0,442	-0,038
PNI	0,130	0,311	0,491	-0,032	0,294	**0,556***
LVI	-0,387	0,387	0,301	0,258	0,301	0,387
PTV	-0,346	-0,083	0,049	-0,419	0,299	0,199
PM	-0,389	-0,272	-0,330	**-0,467***	-0,019	-0,311
